# Ion Adsorption and Desorption at the CaF_2_‐Water Interface Probed by Flow Experiments and Vibrational Spectroscopy

**DOI:** 10.1002/anie.202207017

**Published:** 2022-10-13

**Authors:** Patrick Ober, Johannes Hunger, Sophia H. Kolbinger, Ellen H. G. Backus, Mischa Bonn

**Affiliations:** ^1^ Department of Molecular Spectroscopy Max Planck Institute for Polymer Research Ackermannweg 10 55128 Mainz Germany; ^2^ University of Vienna Faculty of Chemistry Institute of Physical Chemistry Waehringer Strasse 42 1090 Vienna Austria

**Keywords:** Adsorption, Flow, Mineral-Water Interface, Non-Linear Spectroscopy, Vibrational Spectroscopy

## Abstract

The dissolution of minerals in contact with water plays a crucial role in geochemistry. However, obtaining molecular insight into interfacial chemistry is challenging. Dissolution typically involves the release of ions from the surface, giving rise to a charged mineral surface. This charge affects the interfacial water arrangement, which can be investigated by surface‐specific vibrational Sum Frequency Generation (v‐SFG) spectroscopy. For the fluorite‐water interface, recent spectroscopic studies concluded that fluoride adsorption/desorption determines the surface charge, which contrasts zeta potential measurements assigning this role to the calcium ion. By combining v‐SFG spectroscopy and flow experiments with systematically suppressed dissolution, we uncover the interplay of dominant fluoride and weak calcium adsorption/desorption, resolving the controversy in the literature. We infer the calcium contribution to be orders of magnitude smaller, emphasizing the sensitivity of our approach.

## Graphical abstract

Uncovering Ion Adsorption Reactions. The combination of surface‐sensitive v‐SFG spectroscopy and flow experiments allows to uncover the interplay of a dominant fluoride and a weak calcium adsorption reaction at the fluorite‐water interface. This is enabled by systematically changing bulk concentrations and suppression of fluoride dissolution.

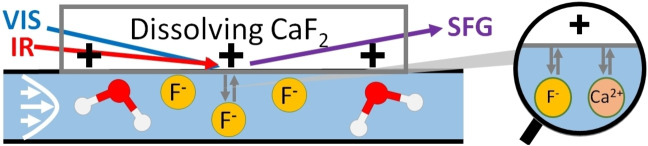

## Introduction

Interfaces are the stage of many important reactions in natural and technical settings. In geochemistry, the omnipresent mineral‐water interface is of particular interest, because reactions at this interface determine mineral growth and dissolution as well as environmental remediation.[^1^, ^2^] To gain molecular insight into mineral‐water interfaces, it is crucial to understand the interplay between chemical reactions, the surface charge, and the structure of interfacial water molecules in addition to ion distributions.[^3^, ^4^] Obtaining such insights is very challenging, as is clear from the common mineral fluorite (i.e., calcium fluoride, CaF_2_). There are numerous studies elaborating on the structure and dynamics of the fluorite‐water interface, its surface potential, streaming potential, and surface chemistry.[^5^, ^6^, ^7^, ^8^, ^9^, ^10^, ^11^, ^12^, ^13^, ^14^, ^15^, ^16^, ^17^] In 2004, Miller et al.^[7]^ employed zeta potential measurements to study how the pH and the presence of fluoride and calcium ions affect fluorite's surface charge. They concluded a potential‐determining role of calcium. Since then, spectroscopic studies have shown substantial interactions of fluoride ions with the fluorite surface.[^12^, ^13^] Most recently, the surface charge and potential of fluorite have been connected to the adsorption/desorption of fluoride, whereas the effect of calcium ions on the surface charge has been neglected.[^9^, ^15^, ^16^, ^17^] Following this simplification, the considered surface chemistry at the bare fluorite‐water interface has remained limited to two reactions: I) Dissolution of calcium fluoride as in Equation (1). II) Adsorption/desorption of fluoride anions as in Equation (2). In these equations, ≡ denotes the solid subphase.

(1) $$\mathrm{Dissolution}\;\mathrm{reaction}:\;\equiv CaF_{2\left(s\right)}\vec{\leftarrow }{Ca}_{\left(aq\right)}^{2+}+{2\;F}_{\left(aq\right)}^{-}$$

(2) $$\mathrm{Fluoride}\;\mathrm{adsorption}/\mathrm{desorption}:\equiv CaF_{2\left(s\right)}\;\vec{\leftarrow }\;\equiv CaF_{\left(s\right)}^{+}+F_{\left(aq\right)}^{-}$$

As both reactions share the fluoride anion, surface charge and dissolution are coupled. A more general interpretation of the coupling between surface charge and dissolution might be that the fluoride desorption from reaction (2) is one step within the overall dissolution process. If so, another step within the dissolution needs to be the desorption of calcium. In this scenario, the interplay of fluoride and calcium desorption would determine the surface charge. This consideration and the controversy in the literature between zeta potential measurements and spectroscopic investigations, raises the question of whether the contributions of fluoride and calcium ions to the surface charge can be disentangled and which ion eventually determines the potential.

As the adsorption/desorption reactions of ions change the surface charge, those reactions can be studied with non‐linear optical spectroscopy. Particularly vibrational Sum Frequency Generation (v‐SFG) spectroscopy is an established method to probe such charged mineral‐water interfaces. In v‐SFG spectroscopy, visible (Vis) and infrared (IR) laser pulses are overlapped in space and time at the interface of interest, generating light at the sum of the two incident frequencies (SFG). This non‐linear optical process is forbidden in centrosymmetric media, such as bulk water. However, at the mineral‐water interface, where the symmetry is broken, SFG light is generated. Resonant enhancement due to tuning a broadband IR pulse to the OH‐stretching frequencies of water confines the sensitivity to broken symmetry to the aqueous phase.[^18^, ^19^] The magnitude of the detected intensity correlates with the net orientation of the water molecules at the interface and the polarization of water molecules due to interfacial fields. Both contributions scale with the surface charge of the mineral, which aligns and polarizes the water molecules due to electrostatic interactions between the water molecules and the electric field arising from the surface charge. Thus, the detected intensity can be used to study surface charges and potentials as well as the interaction of ions and water molecules at a charged surface on a molecular scale.[^4^, ^19^, ^20^, ^21^, ^22^, ^23^, ^24^, ^25^, ^26^, ^27^, ^28^, ^29^]

The v‐SFG intensity $I_{SFG}$ is commonly connected to the surface potential $\Phi_{0}$ by Equation 3.[^21^, ^30^]

(3) $$I_{SFG}\approx {\left|\chi^{\left(2\right)}+\chi^{\left(3\right)}\cdot \Phi_{0}\right|}^{2}$$

Here $\chi^{\left(2\right)}$ and $\chi^{\left(3\right)}$ are the second‐ and third‐order non‐linear susceptibilities, respectively. The surface potential depends on the surface charge and the ionic strength of the aqueous solution, which screens the surface charge, thus limiting the polarization depth. This screening can be described by the Gouy‐Chapman‐model, which leads to Equation (4) for the surface potential.[^21^, ^29^, ^30^, ^31^]

(4) $$\Phi_{0}=\frac{2k_{B}T}{e}\cdot \sinh^{-1}\left(\frac{\sigma }{\sqrt{8k_{B}TN_{A}I\epsilon_{0}\epsilon_{r}}}\right)\;with\;I=\frac{1}{2}\cdot \sum_{i}z_{i}^{2}\;c_{i,bulk}$$

Here, $k_{B}$ is the Boltzmann constant, *T* the temperature, *e* the elementary charge, σ the surface charge density, $N_{A}$ the Avogadro constant, I the ionic strength, $\epsilon_{0}$ the vacuum electric permittivity and $\epsilon_{r}$ the relative permittivity of the present medium (e.g., water), $z_{i}$ the charge of the ion i , and $c_{i,bulk}$ the bulk concentration of that ion.

The sensitivity of the v‐SFG intensity to ion concentrations offers the opportunity to study dissolution processes: The accumulation of ions near the interface due to dissolution when the aqueous phase is at rest, and the concentration decrease due to dilution upon flow have been shown to dramatically affect the surface potential and thus the v‐SFG intensity.[^15^, ^16^, ^20^] Such concentration changes may affect the screening of the surface charge, as well as the surface charge itself, due to the adsorption/desorption equilibria of ions. As a flow‐induced dilution decreases the concentration of adsorbing/desorbing ions and the amount of adsorption/desorption, the surface charge can change. For the fluorite‐water interface, a flow‐induced increase in the positive surface charge has been assigned to a dilution of dissolved fluoride anions and thus reduced adsorption.[^15^, ^16^, ^17^] In this work, we combine v‐SFG spectroscopy and flow experiments to gain additional molecular insights into the surface chemistry at the charged fluorite‐water interface. We use the intensity obtained by v‐SFG spectroscopy as an indirect measure of the surface charge. By systematically suppressing dissolution and flow‐induced concentration changes, we uncover the complex interplay of both fluoride and calcium adsorption/desorption.

## Results and Discussion

We performed experiments with and without applied flow at the fluorite‐water interface under acidic conditions (1 mM HCl) and systematically varied the fluoride bulk concentration by adding different amounts of sodium fluoride to the hydrochloric solution. Under acidic conditions, the adsorption/desorption of fluoride has been connected to the surface charge.[^9^, ^12^, ^15^, ^16^] Yet, to determine at which concentrations added NaF could suppress the influence of fluoride adsorption/desorption, we first study the v‐SFG intensity as a function of added sodium fluoride under static (flow‐off) conditions. To gain additional insights into the dependency of the surface charge on interfacial concentrations of fluoride and possibly calcium, we also investigate how flow‐induced dilution of the dissolved ions affects the v‐SFG intensity.

**Figure 1:**
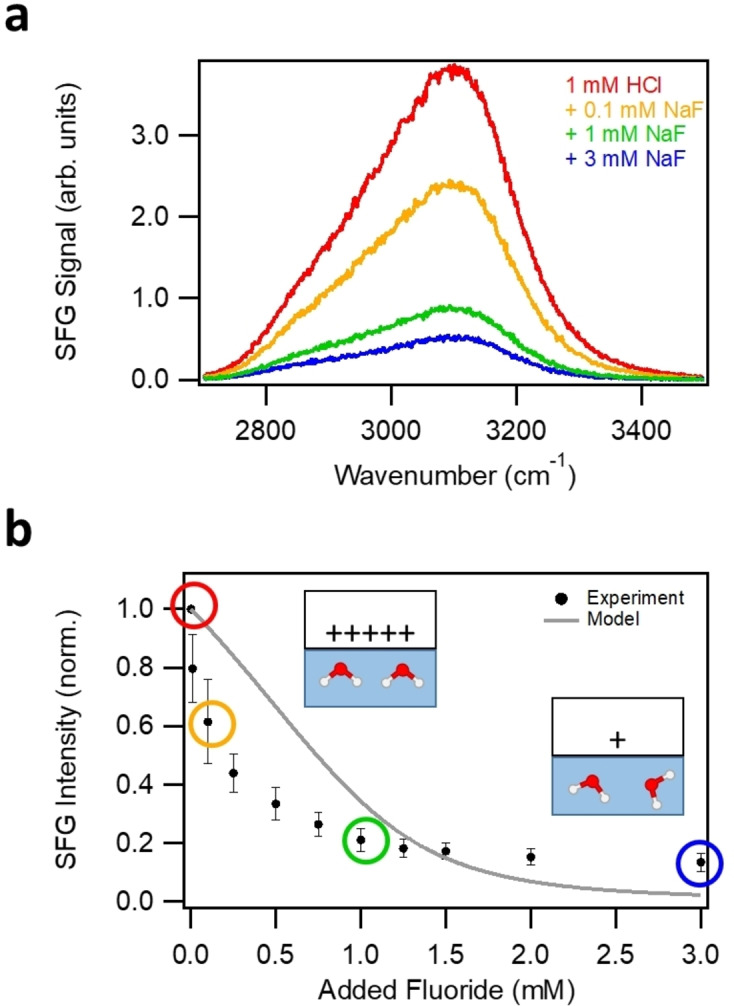
Fluoride‐dependent SFG experiments under flow‐off condition. a) v‐SFG spectra of the fluorite‐water interface with increasing fluoride bulk concentration. b) Dependency of the v‐SFG intensity on the amount of added sodium fluoride. The experimental values (filled black circles) arise from integrating the v‐SFG spectra between 2800 and 3600 cm^−1^. The integrals corresponding to the spectra from Figure 1a are marked by a colored circle with the color matching the color of the spectrum in panel a. The data points are normalized to the bare hydrochloric acid solution, i.e., with no fluoride added to the bulk solution. The error bars arise from several measurements. Inset is an illustration of the decreasing surface charge as the primary cause for the reduced net orientation of the interfacial water molecules resulting in the decreasing v‐SFG intensity. The solid grey line shows our model described in the text.

A selection of v‐SFG spectra for different amounts of added sodium fluoride with the aqueous phase at rest is shown in Figure 1a. Clearly, an increase in fluoride bulk concentration decreases the magnitude of the v‐SFG intensity dramatically. To quantify this decrease, we plotted the integral of the spectra from 2800 to 3600 cm^−1^ as a function of the added sodium fluoride in Figure 1b. The v‐SFG intensity decreases monotonically with the addition of fluoride. This decrease is steeper at lower fluoride concentrations, like ≈20 % between 1 and 10 μ M compared to ≈5 % between 1 and 3 mM. In fact, the intensity seems to level off at mM fluoride concentrations. At the highest NaF concentration, the intensity is only ≈15 % of the intensity without added fluoride (bare 1 mM HCl solution). According to Equations (3) and (4), such a decrease in the v‐SFG intensity, indicative of a lowered surface potential, can have two possible causes: an increase in ionic strength and/or a decreasing surface charge. In the experiments, the addition of sodium fluoride increases the salt concentration and thus the ionic strength, yet also increases the fluoride adsorption, thereby lowering the surface charge according to Equation (2).

**Figure 2:**
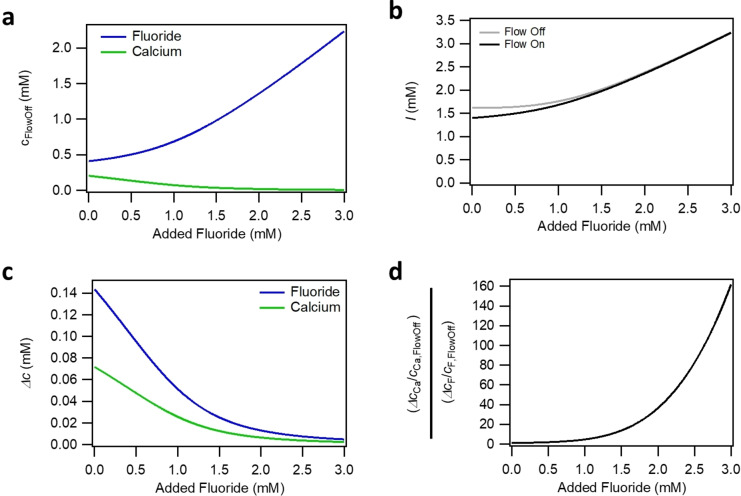
Estimating interfacial concentrations and flow‐induced changes. a) Static (flow‐off) concentration of fluoride anions and calcium cations as a function of added sodium fluoride under the assumption of a saturated flow channel via dissolution of fluorite. b) ionic strength under static flow‐off (grey) and under diluted flow‐on (black) conditions as a function of added sodium fluoride under the assumption of a saturated flow channel via dissolution of fluorite under flow‐off condition and 35 % dilution with the bulk solution from the reservoir upon flow. c) Differences between saturated flow‐off and diluted flow‐on concentrations of fluoride anions and calcium cations as a function of added sodium fluoride $\Delta c=c_{FlowOff}-c_{FlowOn}$ . d) Ratio of relative flow‐induced changes in calcium and fluoride concentration as a function of added sodium fluoride.

In order to estimate how the addition of sodium fluoride to the bulk solution affects the surface charge and the ionic strength, we calculated the interfacial flow‐off ion concentrations $c_{F,FlowOff}$ and $c_{Ca,FlowOff}$ of fluoride and calcium, respectively, as well as the ionic strengths as a function of the added sodium fluoride in Figure 2a and b. Those concentrations are based on the assumption of a saturated flow channel. This means that CaF_2_ dissolves from the interface and diffuses into the flow channel until the solubility product $L_{CaF_{2}}$ is reached according to Equation 5:

(5) $$L_{{CaF}_{2}}=c_{F,FlowOff}^{2}\cdot c_{Ca,FlowOff}={\left(c_{F,bulk}+2x\right)}^{2}\cdot x$$

where x is the concentration of dissolved fluorite. The system is modeled with $L_{CaF_{2}}$ =3.45×10^−2^ mol^3^ m^−9^. The bulk concentrations in the reservoir are assumed to be unaffected by the diffusion and advection of the dissolved ions due to the time scale of the experiment (≈1 h) and the two orders of magnitude smaller volume of the liquid phase in the flow channel as compared to the reservoir. Additionally, the concentration of calcium in the bulk is assumed to be zero. It is clear from Figure 2a that the dissolution, and therefore the concentration of dissolved calcium, decreases with added fluoride. Since we assume a saturated flow channel and since all solutions contain 1 mM HCl, the ionic strength is already in the mM range without sodium fluoride being added to the bulk. Thus, the increase in ionic strength due to added NaF is minor, as evident from Figure 2b. Note that we also consider the minor effect of HF formation as described in the Supporting Information, section S1.1. HF formation leads to small differences between the nominally added concentrations of added NaF and the fluoride bulk concentration.

Considering particularly the most pronounced changes in the v‐SFG intensity up to 0.1 mM added sodium fluoride, there is hardly any change in the ionic strength (grey line in Figure 2b). Therefore, at least for low amounts of added sodium fluoride, the decrease in surface charge due to fluoride adsorption dominates our observations. The connection between surface charge and fluoride concentration can be explained by Equation (2). At elevated fluoride concentrations, the adsorption/desorption equilibrium is shifted to the left side (adsorption), resulting in a lower surface charge density. Consequently, the presented dependency of the v‐SFG intensity on the fluoride concentration is consistent with earlier spectroscopic studies and their conclusion that adsorption/desorption of fluoride anions affects the surface charge and potential.[^9^, ^15^, ^16^, ^17^]

**Figure 3:**
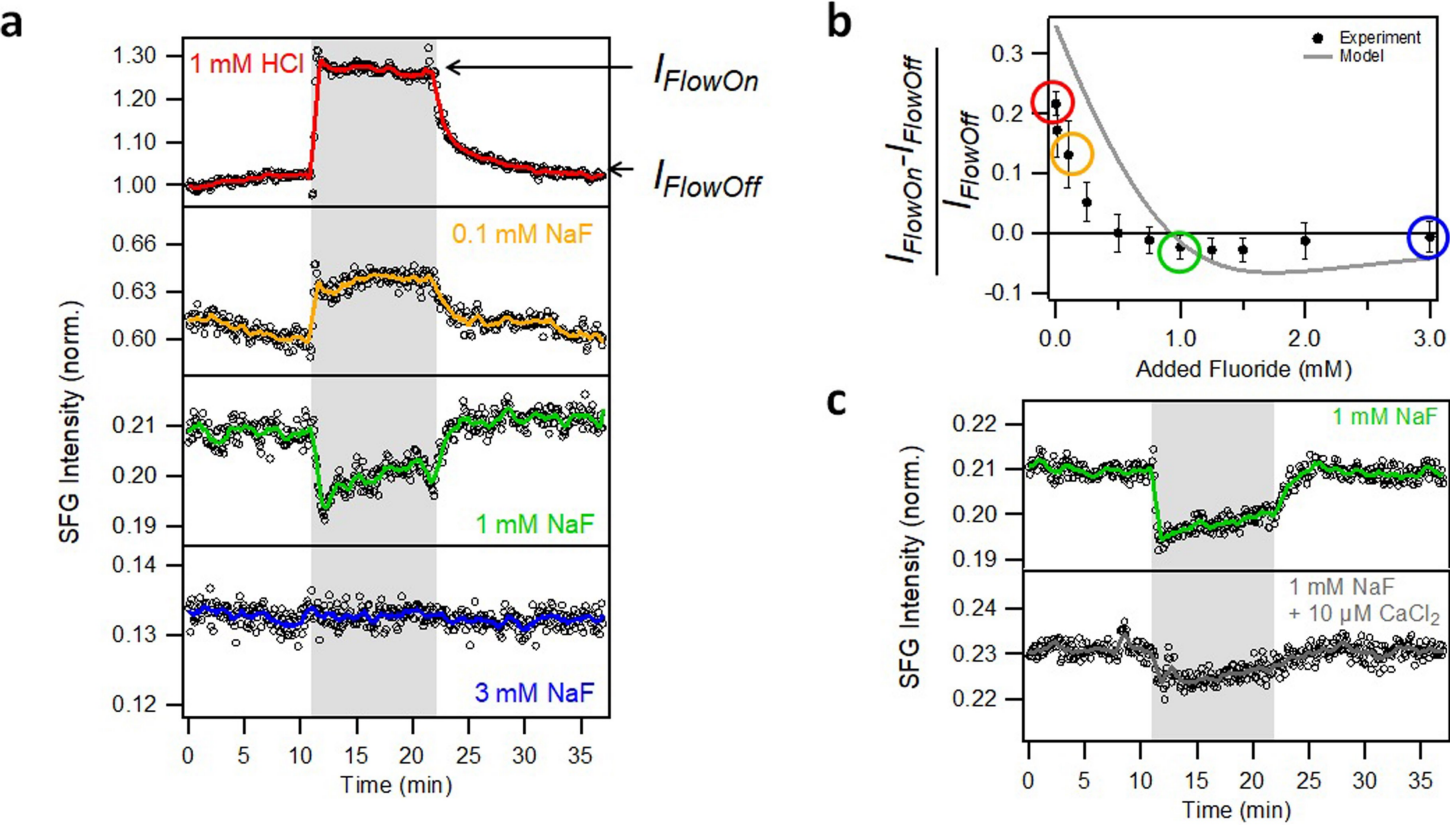
Fluoride‐dependent SFG experiments under flow‐on conditions. a) v‐SFG intensity development during a flow‐on/off cycle. The grey area highlights the flow‐on region with a flow rate of 6 mL min^−1^, corresponding to a Reynolds number of ≈25. Each open black circle represents one integrated v‐SFG spectrum. The colored solid lines are ten‐point moving averages to guide the eye, and the same color is used to mark the corresponding data in Figure 1. The v‐SFG intensities under flow‐off conditions are normalized as in Figure 1a. In the top trace, the level of the v‐SFG intensity at flow‐on and flow‐off conditions is marked by arrows with $I_{FlowOn}$ and $I_{FlowOff}$ , respectively. Quantitatively, the values are averaged over ≈1 min prior to switching between flow‐on/off. b) Plot of the relative change in the v‐SFG intensity induced by flow $\left(I_{FlowOn}-I_{FlowOff}\right)/I_{FlowOff}$ as function of the added sodium fluoride. The filled circles represent the experimental values obtained from the flow cycles. The error bars arise from several flow‐on/off cycles recorded on different days. The relative changes corresponding to the intensity developments from Figure 3a are marked by matching colored circles. The solid grey line is our model as described in the text. c) Same as a) but comparing the absence and presence of 10 μ M added calcium chloride to a 1 mM sodium fluoride and 1 mM HCl solution used for the flow experiment.

To this end, the spectroscopic observations can, at least qualitatively, be explained by fluoride adsorption. To challenge this explanation with additional experiments, we also investigated how the flow‐induced change in the concentration of the dissolved ions affects the v‐SFG intensity as a function of the added amount of NaF. A selection of the v‐SFG intensities during one flow‐on/off cycle for certain sodium fluoride bulk concentrations is shown in Figure 3a, where each open circle represents one integrated spectrum. It becomes clear that the presence of flow can change the v‐SFG intensity. While the v‐SFG intensity increases upon flow at low fluoride bulk concentrations (e.g., bare hydrochloric solution, red line in Figure 3a), the intensity stays constant at high fluoride concentrations (i.e., upon the addition of 3 mM NaF, blue line in Figure 3a). Those observations have been reported in previous studies[^15^, ^16^, ^17^] and can be related to flow‐induced changes in the near‐surface concentration of fluoride ions due to dilution with the fresh solution upon flow. At low fluoride bulk concentrations, the chemical equilibrium in the adsorption/desorption reaction from Equation (2) is shifted to the right side with the higher surface charge due to the decrease in fluoride concentration upon flow‐induced dilution. In contrast, at high fluoride concentrations, the change in concentration upon flow will be minor. Consequently, at 3 mM added NaF, the surface charge does not change significantly upon flow. Interestingly, we observe for intermediate fluoride bulk concentrations (around 1 mM added sodium fluoride, green line in Figure 3a) that the v‐SFG intensity decreases upon flow. To quantify the flow‐induced changes, we average the intensities from ≈1 min prior to switching between flow‐off to flow‐on and vice versa ($I_{FlowOff}$ and $I_{FlowOn}$ ), and plot the relative increase $\left(I_{FlowOn}-I_{FlowOff}\right)/I_{FlowOff}$ as a function of the added amount of NaF in Figure 3b. From this plot, it is clear that the flow‐induced decrease in the v‐SFG intensity is a significant and reproducible observation for a range of concentrations around 1 mM of added NaF.

To understand the change in sign of the flow‐induced surface charge alterations around 1 mM added sodium fluoride, it is instructive to consider the flow‐induced changes in the near‐surface concentrations. While we assume a saturated solution under static conditions (flow‐off), the presence of flow dilutes the solution in the flow channel with the bulk solution from the reservoir. Therefore, the concentration of any ion upon flow $c_{FlowOn}$ must be between the flow‐off concentration $c_{FlowOff}$ and the bulk concentration. The flow‐on condition is incorporated by an effective dilution δ as in Equation 6.

(6) $$c_{FlowOn}=c_{FlowOff}\cdot \left(1-\delta \right)+c_{bulk}\cdot \delta$$

Following numerical simulations of a comparable flow channel, we set δ=0.35 .^[16]^ The differences in concentration Δc between $c_{FlowOn}$ and $c_{FlowOff}$ for calcium and fluoride ions is shown as flow‐induced concentration changes as a function of the added sodium fluoride in Figure 2c.

Compared to the flow‐induced changes in the concentrations of fluoride and calcium ions, the ionic strength is only altered slightly by the presence of flow (see Figure 2b), which explains why the concentration changes of adsorbing ions and thus the surface charge changes dominate the surface potential changes. Yet, upon the addition of NaF, the saturated flow‐off calcium and fluoride concentrations do not obey 1 : 2 stoichiometry. As such, calcium‘s relative dilution becomes more prominent compared to fluoride. This is illustrated in Figure 2d, where we relate the relative flow‐induced concentration changes $\Delta c/c_{Off}$ of calcium ions to those of the fluoride ions. As this value increases with the added amount of sodium fluoride, the dilution of calcium ion concentration upon flow becomes more relevant with increased fluoride bulk concentrations. To understand how this subtle interplay between flow‐induced concentration changes impacts the changed sign in the v‐SFG intensity change upon flow, we propose that not only fluoride but also calcium adsorption/desorption (equation (7)) needs to be considered – in particular at intermediate NaF concentrations.

(7) $$\mathrm{Calcium}\;\mathrm{adsorption}/\mathrm{desorption}:\equiv CaF_{2\left(s\right)}+{Ca}_{\left(aq\right)}^{2+}\vec{\leftarrow }\;\equiv {Ca}_{\left(s\right)}^{2+}$$

Here, the adsorption of a calcium ion to a terminal uncharged $\equiv CaF_{2}$ site gives rise to a terminal $\equiv Ca^{2+}$ site. Accordingly, the interplay of flow‐induced fluoride and calcium adsorption/desorption can make the surface charge more positive or less positive. Since with increasing NaF concentration, the effect of flow‐induced changes to the calcium concentration becomes more important (Figure 2d), its impact on the surface charge [Eq. (7)] overwhelms the effect of flow‐induced changes in the fluoride concentration on the surface charge [Eq. (2)]. This explains the observation of the different signs of the flow‐induced change in the v‐SFG intensity in Figure 3.

To confirm the contribution of calcium adsorption/desorption, we performed additional flow experiments with and without added calcium. If the observed decrease in the v‐SFG intensity upon flow is due to a dilution in calcium concentration, indicating calcium adsorption/desorption, the addition of calcium to the bulk will lower the dilution of calcium upon flow. Consequently, the change in surface charge and the corresponding v‐SFG intensity will become less pronounced when calcium is added to the bulk. Figure 3c shows the results for flow experiments of 1 mM HCl with 1 mM NaF solutions with and without the addition of 10 μ M CaCl_2_. Clearly, the flow‐induced change in the v‐SFG intensity becomes smaller when 10 μ M CaCl_2_ is added. We can thus conclude that both fluoride and calcium adsorption/desorption contribute to the surface charge by the two reactions [Eqs. (2) and (7)] in a competitive manner. Upon flow, the two reactions have opposite effects: the flow‐induced reduction of the interfacial fluoride concentration increases the surface charge by driving the equilibrium from Equation (2) to the right side. The dilution in calcium concentration decreases the surface charge by driving the equilibrium from Equation (7) to the left side. At 1 mM fluoride bulk concentration, we suppress the effect of fluoride adsorption/desorption on the dilution upon flow, revealing the calcium contribution. Since the addition of fluoride suppresses the dissolution process, also the calcium concentration and dilution become smaller with increasing fluoride concentrations (Figure 2a and c), which is why at 3 mM of added sodium fluoride there is no more change upon flow in the adsorption/desorption equilibria and thus in surface charge and observed v‐SFG intensity.

The effect of calcium adsorption on the surface charge is further supported by the magnitude of the v‐SFG intensity in the flow‐off state. From comparing the two data sets in Figure 3c, it is clear that the addition of calcium increases the surface charge, as one can see from the ≈10 % higher v‐SFG intensity under flow‐off condition.

Based on the evidence for an interplay of fluoride and calcium adsorption/desorption at the fluorite‐water interface, we aim to compare the contributions of those two reactions quantitatively. Clearly, the fluoride adsorption/desorption reactions dominate, which causes the increase in the surface charge and v‐SFG intensity upon flow when no fluoride is added. To estimate the ratio between fluoride and calcium adsorption/desorption equilibria, we model the experimental data based on Equations (1)–(7). The adsorption/desorption equilibria for fluoride and calcium, which determine the surface charge, are incorporated via a simple Langmuir model. In such a model, the adsorption of a particle A to the surface S increases with the interfacial concentration (under either flow‐off or flow‐on condition) of A, $c_{A}$ , but is limited by the density of adsorption/desorption sites Γ to a monolayer as in Equation (8). Here the amount of adsorption is quantified by the density of occupied adsorption sites $\Gamma_{SA}$ . The strength of adsorption depends on the rate constants for adsorption and desorption $k_{ads}$ and $k_{des}$ , respectively. Note that the Langmuir model neglects any interaction between different surface sites as well as electrostatic interactions. Details on the mathematical background and an extension of Equation (8) towards the adsorption/desorption of two species (calcium and fluoride ions) are presented in the Supporting Information, section S1.2.

(8) $$\Gamma_{SA}=\frac{\Gamma }{1+k_{des}/k_{ads}\cdot \frac{1}{c_{A}}}\;for\;the\;reaction\;S+A\vec{\leftarrow }SA$$

According to Equation (8) as well as the extension from equation (S‐15), the modeling requires knowledge of the concentration of the adsorbing/desorbing ions under flow‐off and flow‐on, which were discussed above and presented in Figure 2. The data that we modeled is the normalized v‐SFG intensities from Figure 1b and their flow‐induced change $\left(I_{FlowOn}-I_{FlowOff}\right)/I_{FlowOff}$ in Figure 3b. The experimental data and the model are shown together in those figures. As further described in the Supporting Information, section S1.3, we used, in the model, Γ=10^19^ m^−2^, $L_{CaF_{2}}$ =3.45×10^−2^ mol^3^ m^−9^, and 35 % dilution upon flow. The parameters $\chi^{\left(3\right)}/\chi^{\left(2\right)}=$ 250 V^−1^ and the rate constants $k_{ads,F}/k_{des,F}=$ 700 m^3^ mol^−1^ for fluoride and $\frac{k_{ads,Ca}}{k_{des,Ca}}=$ 0.005 m^3^ mol^−1^ for calcium adsorption/desorption were chosen to describe best the experimental data and to match the surface potential at zero added sodium fluoride with the reported ζ ‐potential of ≈70 mV.^[7]^ Also, a slight pH dependency accounting for the HF formation is incorporated, see Figure S1 in the SI. Despite its simplicity, our model captures the main behavior of the experimental data. This behavior is the monotonic decrease in the static v‐SFG intensities and the transition from a flow‐induced increase in the v‐SFG intensity at low fluoride concentrations to a flow‐induced decrease at intermediate concentrations and no change at high fluoride concentrations. Particularly the flow experiments provide a sensitive part within the modeling towards the calcium contribution as we emphasize in Figure S2 in the SI, where we compare our extended model with one that considers the adsorption/desorption of fluoride anions only.

We want to emphasize that our model gives rather conceptual insights and that quantitative values of the adsorption rate constants should be handled with caution. This is because our simple model misses some quantitative features of the experimental data, such as the steep decrease in the v‐SFG intensity at low fluoride concentrations or the underestimation of the level of the plateau in the mM range for the flow‐off intensities (Figure 1b). Additionally, the model shows a shifted and stretched curve of the flow‐induced changes (Figure 3b). All those deviations might be connected to the assumed charge independence of $\chi^{\left(2\right)}$ , the limitations of the Gouy‐Chapman‐model at higher surface potentials, the neglect of electrostatics, or other simplifications within the Langmuir adsorption model. Moreover, the discrepancies are presumably related to the fact that changes in the SFG intensities evidence relative variations in surface charge, while in the model, absolute surface charge values are required, making the obtained parameters somewhat ambiguous: the description of the flow‐off data is slightly insensitive to the exact choice of the model parameters (Figure S3a in the SI). However, we show in Figure S3 in the SI that particularly the magnitude of the predicted flow‐induced changes is very sensitive to the ratio of the adsorption equilibria for calcium and fluoride: If these ratios are altered by a factor of 5, the theoretically predicted flow‐induced v‐SFG changes markedly over/under‐estimate the experimental observations and miss key features as the change in the sign of the flow‐induced intensity changes. As such, the flow experiments displayed in Figure 3 are very sensitive to the relative adsorption equilibria of both ions: Based on the model, we find a clear dominance of the fluoride adsorption/desorption by comparing the used rate constants from our model $(\frac{k_{ads,F}}{k_{des,F}}/\frac{k_{ads,Ca}}{k_{des,Ca}}$ ). From this comparison, the fluoride adsorption/desorption exceeds that of calcium by ≈5 orders of magnitude. Since our modeling is based on several assumptions and simplifications like the Langmuir adsorption model, we mainly aim to gain mechanistic insights. The experimental results in conjunction with the model clearly support the interplay of two adsorption reactions, with a vast predominance of fluoride dissolution over calcium. While quantitative adsorption rates are challenging to obtain, the dominance of fluoride adsorption by several orders of magnitude can be firmly concluded. We also use our estimation to discuss the impact of fluoride and calcium concentrations on the surface charge (Figure S4a in the SI), and at which added fluoride concentrations a change in concentration as in the flow experiments is dominated by which adsorption process. We also apply our model to estimate the present range of surface charges in Figure S4b in the SI. It turns out that throughout all considered concentrations in the experiment, less than 1 % of the surface sites are charged due to the dominant adsorption of fluoride anions. Therefore, the concentration‐ and flow‐dependent v‐SFG intensity variations reflect only a very small range of surface charge variations, suggesting a high sensitivity of the experiments.

The estimated dominance of fluoride adsorption/desorption makes it remarkable that the small contribution of calcium can eventually determine the flow‐induced changes at a certain fluoride bulk concentration. With this sensitive experimental approach of combining surface‐specific v‐SFG spectroscopy and flow experiments, we uncovered and quantified the complex interplay of a dominant fluoride and a weak calcium adsorption/desorption reaction. This resolves the controversy in literature regarding the potential‐determining role of the two ions: Fluoride adsorption is orders of magnitude more dominant; however, there is a calcium contribution, which can determine surface charge changes upon concentration changes as probed in the flow experiment. We expect that the combination of flow experiments and surface‐sensitive spectroscopy can be a useful tool for studying interfacial reactions over a wider range of systems. Such an experiment requires the spectroscopic accessibility of the surface and a concentration gradient between the interface where reactions occur and the bulk phase as the presence of flow needs to alter interfacial concentrations and thus chemical equilibria of the (even indirect) spectroscopically investigated species. Applying our approach should be straightforward for other mineral‐water interfaces, but could also prove to be insightful in other fields of surface chemistry like heterogeneous catalysis or electrochemistry.

## Conclusion

In this work, we have demonstrated that the combination of surface‐specific v‐SFG spectroscopy and flow experiments is suitable for uncovering the complex surface chemistry of adsorption/desorption reactions at the fluorite‐water interface. Due to the high sensitivity of our approach, we revealed that calcium adsorption/desorption is a small contribution to the surface charge in addition to the dominant fluoride adsorption/desorption. Based on v‐SFG experiments with and without applied flow for a series of fluoride concentrations, we suppressed the contribution of fluoride adsorption/desorption to the flow‐induced alteration in the chemical equilibrium at the interface. We verified our hypothesis by comparing experiments at one fluoride concentration with and without the addition of only 1 % calcium in the bulk relative to fluoride. Our interpretation is further supported by a simple model based on two competing Langmuir adsorption/desorption reactions, which conceptually confirmed the interplay of two adsorption reactions and allowed us to estimate the dominance of the fluoride adsorption/desorption reaction: the effect of fluoride on the surface charge is several orders of magnitude stronger than the effect of calcium. With those findings, we resolve a controversy in literature that so far considered only one of the two adsorption/desorption reactions but not the interplay of both. Overall, the combination of surface‐specific v‐SFG spectroscopy to indirectly measure the surface charge and flow experiments can be used to study chemistry at a mineral‐water interface with high sensitivity. It is possible to reveal the interplay between very small contributions and thus the complexity of interfacial reactions. We, therefore, expect that this approach finds its application in future investigations of surface reactions at a variety of solid‐fluid interfaces, particularly in geochemistry.

## Author Contributions

P.O., J.H., E.H.G.B., and M.B. designed the research project. P.O. and S.H.K. performed the experiments, P.O. analyzed the data and developed the model. All authors discussed the results and wrote the manuscript.

## Conflict of interest

The authors declare no conflict of interest.

1

## Supporting information

As a service to our authors and readers, this journal provides supporting information supplied by the authors. Such materials are peer reviewed and may be re‐organized for online delivery, but are not copy‐edited or typeset. Technical support issues arising from supporting information (other than missing files) should be addressed to the authors.

Supporting Information

## Data Availability

The data that support the findings of this study are available from the corresponding author upon reasonable request.
