# Supplementary material for: Ion Adsorption and Desorption at the CaF2‐Water Interface Probed by Flow Experiments and Vibrational Spectroscopy
Source: Angew Chem Int Ed Engl. 2022 Oct 13;61(46):e202207017. doi: 10.1002/anie.202207017 (PMC9828343; doi:10.1002/anie.202207017)
Supplement: Supplementary file 1 — Supporting Information [file ANIE-61-0-s001.pdf]

Angewandte Chemie
Eine Zeitschrift der Gesellschaft Deutscher Chemiker

Supporting Information

Ion Adsorption and Desorption at the CaF_2-Water Interface Probed by Flow Experiments and Vibrational Spectroscopy

P. Ober, J. Hunger, S. H. Kolbinger, E. H. G. Backus, M. Bonn**

S1 Details on the modeling

S1.1. Calculating concentrations and incorporating HF formation.

The interfacial concentrations both under flow-off and flow-on conditions are calculated based on the bulk concentration in the reservoir and the amount of the dissolution into the flow channel as described in the main text. Note, that the bulk concentration is the added amount of NaF minus the amount of formed HF according to equation (S-1). The latter reduces the fluoride and proton concentrations in the bulk.

$$\mathrm{H}^+_{(\mathrm{aq})} + \mathrm{F}^-_{(\mathrm{aq})} \rightleftarrows \mathrm{HF}_{(\mathrm{aq})} \tag{S-1}$$

In chemical equilibrium, equation (S-2) needs to be fulfilled. From this, one can obtain the amount of formed HF $c_{\mathrm{HF,bulk}}$. By using then $c_{\mathrm{F,bulk}} = c_{\mathrm{F,0}} - c_{\mathrm{HF,bulk}}$ the fluoride bulk concentration $c_{\mathrm{F,bulk}}$ after HF formation (equation (S-3)) can be calculated.

$$\mathrm{K_a} = 10^{-\mathrm{pK_a}} = \frac{c_{\mathrm{H,bulk}} \cdot c_{\mathrm{F,bulk}}}{c_{\mathrm{HF,bulk}}} = \frac{\left(c_{\mathrm{H,0}} - c_{\mathrm{HF,bulk}}\right) \cdot \left(c_{\mathrm{F,0}} - c_{\mathrm{HF,bulk}}\right)}{c_{\mathrm{HF,bulk}}} \tag{S-2}$$

$$c_{\mathrm{F,bulk}} = c_{\mathrm{F,0}} - \frac{c_{\mathrm{F,0}} + c_{\mathrm{H,0}} + 10^{-\mathrm{pK_a}}}{2} + \sqrt{\left(\frac{c_{\mathrm{F,0}} + c_{\mathrm{H,0}} + 10^{-\mathrm{pK_a}}}{2}\right)^2 - c_{\mathrm{F,0}} \cdot c_{\mathrm{H,0}}} \tag{S-3}$$

The equations (S-2) and (S-3) involve the concentrations of protons, fluoride anions and formed HF. While, the index zero denotes concentrations corresponding to the added amount before considering HF formation, the index bulk denotes bulk concentrations after considering HF formation. We take the $\mathrm{pK_a}$ of HF as 3.2.[1] The latter is used for the further calculations (e.g., calculating the amount of dissolution und dilution upon flow). The changes in the bulk fluoride concentration equal the changes in the bulk proton concentration, which allows to calculate the slight increase in the pH as shown in Figure S1a. Such a pH change is known to affect the surface charge at the fluorite-water interface and thus the v-SFG intensity. We account for the slight pH dependency by an empirical correction function f_{corr} based on a linear fit through the decreasing v-SFG intensities from a pH series between pH 3 and 3.5, which is approximately the pH range of our experiments. Those data are shown in Figure S1b.

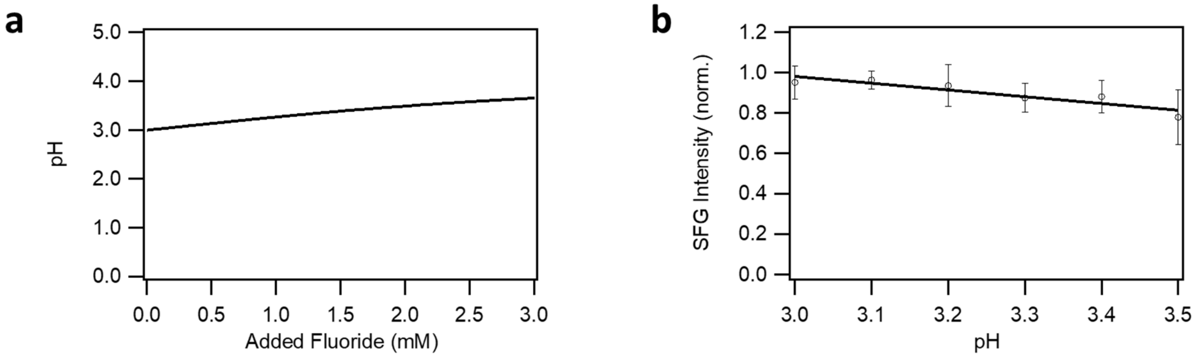

Figure S1 Correcting for pH changes upon HF formation. a) pH as a function of added sodium fluoride. The change is due to the formation of HF from the protons (initially pH 3) and the added sodium fluoride. b) v-SFG intensity as a function of pH. The pH is set by diluting a hydrochloric acid (HCl) solution. The values are based on three independent series where the solutions were measured in the order of increasing pH at least two times in a row. The data point for the pH 3 solution was missing for one series, so the series are normalized to the corresponding pH 3.1 intensity of each first round. The set of the normalized intensities are averaged and their standard deviation are shown as error bars. The solid black line is a linear fit, which, normalized then to pH 3, is used as a correction to incorporate the pH dependency in our modeling.

S1.2. Mathematical description of adsorption/desorption reactions

S1.2.1. Fluoride adsorption/desorption only

For didactic purposes, we elaborate first on the adsorption/desorption equilibrium of one species only, which, for now, is the fluoride anion. The equations (S-4)-(S-8) show the derivation of the surface charge (density) σ of calcium fluoride as a function of the interfacial fluoride concentration c_{F}. Depending on whether the surface charge under flow-off or flow-on conditions needs to be calculated, the corresponding concentration is used. The derivation is the application of the Langmuir adsorption model to the fluorite-water interface, neglecting electrostatic interactions. We are considering the adsorption reaction from equation (S-4). According to this reaction, a surface site can be either uncharged ($\equiv \mathrm{CaF_2}$) or singly positively charged ($\equiv \mathrm{CaF^+}$). Thus, the total density of sites Γ is split according to equation (S-5). The density of charged sites multiplied with the elementary charge e equals the surface charge (density) σ as in equation (S-6).

$$\equiv \mathrm{CaF_2} \rightleftarrows \equiv \mathrm{CaF^+} + \mathrm{F^-} \qquad \text{(S-4)}$$

$$\Gamma = \Gamma_{\equiv CaF_2} + \Gamma_{\equiv CaF^+} \qquad \text{(S-5)}$$

$$\sigma = e\, \Gamma_{\equiv CaF^+} \qquad \text{(S-6)}$$

In a steady state, the time derivative of the surface charge $d\sigma/dt$ is zero (equation (S-7)), and the adsorption and desorption reactions are in equilibrium. Experimentally, this is observed by a constant v-SFG response over time. The equilibrium surface charge is then given by equation (S-8).

$$\frac{d\sigma}{dt} = e\,(-k_{\mathrm{ads,F}}\, c_{\mathrm{F}}\, \mathit{\Gamma}_{\mathrm{CaF^+}} + k_{\mathrm{des,F}}\, \mathit{\Gamma}_{\mathrm{CaF_2}}) = 0 \qquad \text{(S-7)}$$

$$\sigma = \frac{e\,\Gamma}{1 + \frac{k_{\mathrm{ads,F}}}{k_{\mathrm{des,F}}}\,c_{\mathrm{F}}} \qquad \text{(S-8)}$$

S1.2.2. Fluoride and calcium adsorption/desorption

As discussed in the main text, we have to consider not only the adsorption/desorption of fluoride but also of calcium, as described in equation (S-9). We assume a competition between the reactions (S-4) and (S-9). Thus, a surface site can be uncharged ($\equiv \mathrm{CaF_2}$), singly positively charged ($\equiv \mathrm{CaF^+}$) or doubly positively charged ($\equiv \mathrm{Ca^{2+}}$). Therefore, equations (S-5)-(S-7) turn into equation (S-10)-(S-12). Note that in equation (S-9), the uncharged terminal CaF_2 site can be considered part of the solid subphase ($\equiv$) when a calcium ion adsorbs. Conversely, once a terminal Ca^{2+} group is desorbed, the underlying CaF_2 unit from the solid phase becomes the terminal surface site, which is uncharged.

$$\equiv \mathrm{CaF_2} + \mathrm{Ca^{2+}} \rightleftarrows\, \equiv \mathrm{Ca^{2+}} \qquad \text{(S-9)}$$

$$\Gamma = \Gamma_{\equiv\mathrm{CaF_2}} + \Gamma_{\equiv\mathrm{CaF^+}} + \Gamma_{\equiv\mathrm{Ca^{2+}}} \qquad \text{(S-10)}$$

$$\sigma = e\,(\Gamma_{\equiv\mathrm{CaF^+}} + 2\,\Gamma_{\equiv\mathrm{Ca^{2+}}}) \qquad \text{(S-11)}$$

$$\frac{d\sigma}{dt} = e\left(-k_{\mathrm{ads,F}}\,c_F\,\Gamma_{\equiv\mathrm{CaF^+}} + k_{\mathrm{des,F}}\,\Gamma_{\equiv CaF_2} + 2\,k_{\mathrm{ads,Ca}}\,c_{\mathrm{Ca}}\,\Gamma_{\equiv\mathrm{CaF_2}} - 2\,k_{\mathrm{des,Ca}}\,\Gamma_{\equiv\mathrm{Ca^{2+}}}\right) = 0 \qquad \text{(S-12)}$$

The limitation to the three possible surface species ($\equiv \mathrm{CaF_2}$, $\equiv \mathrm{CaF^+}$, $\equiv \mathrm{Ca^{2+}}$) is consistent with the unchanged spectral shape of the v-SFG spectra upon the addition of fluoride as shown in the main text. Those spectral shapes suggest the surface charge to be positive throughout. If there was a change towards a net negatively charged surface, one would see a clear shift of the OH-stretch band in the v-SFG spectra as well as a minimum in the intensities when passing the point of zero charge.[2] However, the v-SFG spectra report only on an average orientation of the water molecules. Therefore, only information on an average net charge is accessible, which is why it might still be possible that additional reactions are present and other surface species are also possible. Such a possibility could be fluoride adsorption at an uncharged site, leading to a singly negatively charged site, like ($\equiv \mathrm{F^-}$). Moreover, a direct conversion between a one times positively charged ($\equiv \mathrm{CaF^+}$) and two times positively charged ($\equiv \mathrm{Ca^{2+}}$) surface site via another fluoride adsorption/desorption reaction would be plausible. Such additional reactions or surface sites are neglected for simplicity.

In equilibrium, adsorption and desorption are balanced, and the number of the different surface sites stays constant, which leads to the equations (S-13) and (S-14).

$$\frac{d\Gamma_{\equiv CaF^+}}{\mathrm{dt}} = -k_{\mathrm{ads,F}}\,c_{\mathrm{F}}\,\Gamma_{\mathrm{CaF^+}} + k_{\mathrm{des,F}}\,\Gamma_{\mathrm{CaF_2}} = 0 \qquad \text{(S-13)}$$

$$\frac{d\Gamma_{\equiv Ca^{2+}}}{\mathrm{dt}} = k_{\mathrm{ads,Ca}}\,c_{\mathrm{Ca}}\,\Gamma_{\equiv CaF_2} - k_{\mathrm{des,Ca}}\,\Gamma_{\equiv\mathrm{Ca^{2+}}} = 0 \qquad \text{(S-14)}$$

The equations (S-10)-(S-14) form a set of linear equations. Solving this for the surface charge σ leads to equation (S-15), which we used for the model in the main text.

$$\sigma = \frac{\mathrm{e}\,\Gamma}{1 + \frac{k_{\mathrm{ads,F}}}{k_{\mathrm{des,F}}}\,c_F + \frac{k_{\mathrm{ads,F}}}{k_{\mathrm{des,F}}}\,\frac{k_{\mathrm{ads,Ca}}}{k_{\mathrm{des,Ca}}}\,c_{\mathrm{F}}\,c_{\mathrm{Ca}}} + \frac{2\,e\,\Gamma}{1 + \frac{k_{\mathrm{des,Ca}}}{k_{\mathrm{ads,Ca}}}\,\frac{1}{c_{\mathrm{Ca}}} + \frac{k_{\mathrm{des,Ca}}}{k_{\mathrm{ads,Ca}}}\,\frac{1}{c_{\mathrm{Ca}}}\,\frac{k_{\mathrm{des,F}}}{k_{\mathrm{ads,F}}}\,\frac{1}{c_{\mathrm{F}}}} \qquad \text{(S-15)}$$

S1.3. Describing Experimental Data

From the added fluoride amount $c_{\mathrm{F,0}}$, the fluoride bulk concentrations after HF formation (subsection S1.1), the interfacial concentrations under saturated flow-off and diluted flow-on conditions are calculated taking into account the dissolution of fluorite and flow-induced dilution as described in the main text (equations (5) and (6)). With those concentrations the surface charge is calculated according to the Langmuir adsorption/desorption of fluoride and calcium (equation (S-15)). The surface charge is then converted to the surface potential and subsequently to a v-SFG intensity according to equations (3) and (4) of the main text. Note that the concentrations in the flow channel were used instead of bulk concentrations to calculate the ionic strength as this is the concentration in the aqueous phase which screens the surface charge of the mineral. For a series of fluoride addition $c_{\mathrm{F,0}}$ the flow-off intensities are normalized to the one with zero added fluoride ($c_{\mathrm{F,0}} = 0$). Thus, the flow-off intensities were modeled according to equation (S-16), using a charge-independent $\chi^{(2)}/\chi^{(3)}$-contribution as fit parameter. Additionally, a pH-dependency of the v-SFG intensities is by an empirical correction function f_{corr} based on a linear fit through the decreasing v-SFG intensities from a pH series (Figure S1b).

$$I_{\mathrm{norm},i} = \frac{\left(\chi^{(2)}/\chi^{(3)} + \Phi_{0,\mathrm{FlowOff}}\left(c_{\mathrm{F,0}}\right)\right)^2}{\left(\chi^{(2)}/\chi^{(3)} + \Phi_{0,\mathrm{FlowOff}}\left(c_{\mathrm{F,0}} = 0\right)\right)^2} f_{\mathrm{corr}}\left(\mathrm{pH}\left(c_{\mathrm{F,0}}\right)\right) \qquad \text{(S-16)}$$

Similarly, the flow-induced changes are modeled with equation (S-17), which relates the flow-on to the flow-off intensities at a given amount of added fluoride $c_{\mathrm{F,0}}$.

$$\left(\frac{I_{\mathrm{FlowOn}} - I_{\mathrm{FlowOff}}}{I_{\mathrm{FlowOff}}}\right)_i = \frac{\left(\chi^{(2)}/\chi^{(3)} + \Phi_{0,\mathrm{FlowOn}}(c_{\mathrm{F,0}})\right)^2}{\left(\chi^{(2)}/\chi^{(3)} + \Phi_{0,\mathrm{FlowOff}}(c_{\mathrm{F,0}})\right)^2} - 1 \qquad \text{(S-17)}$$

For modeling, $\Gamma = 10\ \mathrm{nm}^{-2}$ is taken as the total density of surface sites.[3] The ratio of the rate constants for both fluoride adsorption/desorption as well as calcium adsorption/desorption were adjusted to describe the experimental data and to match the reported surface potential of 70 mV at pH 3 with zero added fluoride.[4] The last step of modeling was to optimize the $\chi^{(2)}/\chi^{(3)}$ ratio within the limitation of a positive sign. For the discussion in the main text and section S3, the inverse ($\chi^{(3)}/\chi^{(2)}$) is referred to since this value is more common in literature. The best model of the data and the surface potential discussed in the main text is obtained using $k_{\mathrm{ads,F}}/k_{\mathrm{des,F}} = 700$ m^3·mol^{-1}, $k_{\mathrm{ads,Ca}}/k_{\mathrm{des,Ca}} = 0.005$ m^3·mol^{-1}, and $\chi^{(3)}/\chi^{(2)} = 250\ \mathrm{V}^{-1}$.

S2 Sensitivity of the flow experiment towards the calcium ion adsorption

We want to emphasize the sensitivity of the flow experiment towards the calcium ion adsorption by comparing our model with a simplified one that only considers fluoride adsorption/desorption. Figure S2 shows the experimental data (closed black circles) and the result of the two models. The models use the same flow-on and flow-off concentrations for fluoride and calcium as interfacial concentration to calculate surface charges and surface potentials. For the simpler model, we set the rate constant $k_{\mathrm{ads,F}}/k_{\mathrm{des,F}} = 440\ \mathrm{m}^3 \cdot \mathrm{mol}^{-1}$ in order to match the experimental value of the surface potential with 70 mV.[4] This simpler model was optimized with $\chi^{(3)}/\chi^{(2)} = \infty\ \mathrm{V}^{-1}$, which is zero $\chi^{(2)}$.

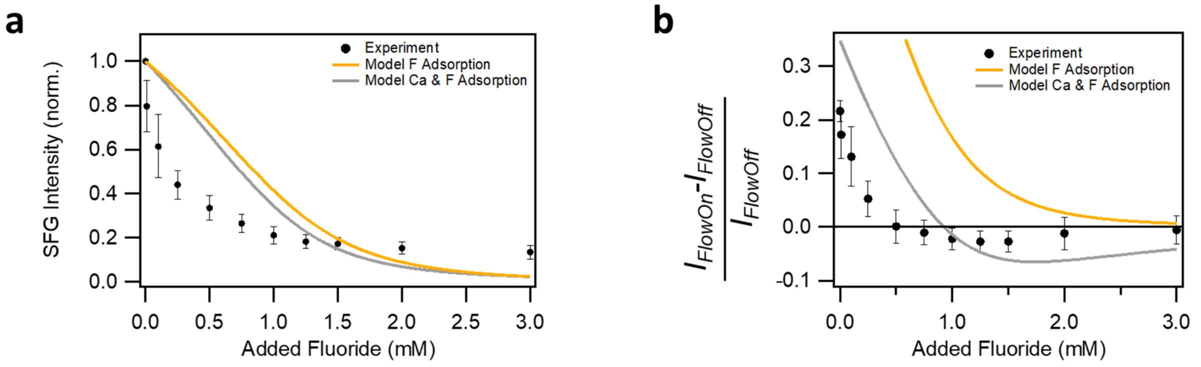

Figure S2. Comparison of the experimental data and the models with only fluoride adsorption/desorption (orange) and the model with both calcium and fluoride adsorption/desorption (grey). a) v-SFG intensity as a function of added sodium fluoride as in Figure 1b of the main text. b) Plot of the relative change in the v-SFG intensity induced by flow as a function of the added sodium fluoride as in Figure 3b of the main text.

From comparing the two models, it is clear that the one with including both calcium and fluoride adsorption/desorption describes the data better than the model with only including adsorption/desorption of fluoride. Both models have their quantitative weaknesses and capture only the main trend of the static (flow-off) v-SFG intensities from Figure S2a, which is the monotonic decrease. However, when it comes to the key features of the flow-induced changes in Figure S2b, particularly the negative regime, only the model that considers both fluoride and calcium adsorption/desorption describes this characteristic observation. Additionally, the model that uses the fluoride adsorption only does overestimated the flow-induced increases massively. This emphasizes the high sensitivity of the flow-experiment, that was necessary to establish the second adsorption/desorption reactions.

S3 Discussion of Adsorption/Desorption Rate Constants

We want to emphasize that the purpose of the modeling is to estimate the ratio between fluoride and calcium adsorption/desorption. From our model, the adsorption/desorption rate of fluoride is about five orders of magnitude higher than the one of calcium. To confirm this dominance, we discuss next how a comparably slight variation in those ratios by a factor of five affects the quality of the fit and the description of the surface potential at zero added fluoride in Figure S3 and Table S1.

Table S1 Variation of model parameters. The variation of the rate constants of fluoride and calcium adsorption/desorption leads to different surface potentials and $\chi^{(3)}/\chi^{(2)}$ values for fitting the data as in Figure S3.

	Model	Model with 5 x F^- adsorption	Model with 0.2 x F^- adsorption	Model with 0.2 x Ca^{2+} adsorption	Model with 5 x Ca^{2+} adsorption
$\chi^{(3)}/\chi^{(2)}$ (V^{-1})	250	63	∞	28	51
$k_{ads,F}/k_{des,F}$ (m^3 mol^{-1})	700	3500	140	700	700
$k_{ads,Ca}/k_{des,Ca}$ (m^3 mol^{-1})	0.005	0.005	0.005	0.001	0.025
Φ_0 (mV)	70	42	130	55	113

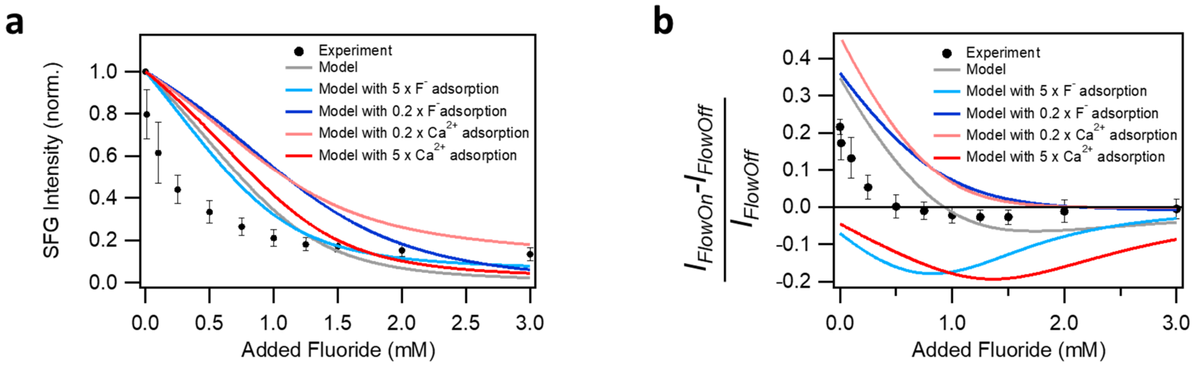

Figure S3.Variation of model parameters. a) v-SFG intensity as a function of added sodium fluoride as in Figure 1b of the main text. The solid lines are based on our model with varying the rate constants according to Table S1. b) Plot of the relative change in the v-SFG intensity induced by flow as a function of the added sodium fluoride as in Figure 3b of the main text. The solid lines are based on our model with varying the rate constants according to Table S1

Our model particularly misses the steep decrease in the v-SFG intensity at low amounts of added sodium fluoride as in Figure S3a and Figure 1b of the main text. The decrease is steeper if the fluoride adsorption rate is increased because this leads to more adsorption of fluoride and thus a steeper decrease in the surface charge. This is consistent with the light blue curve, which describes the experimental data in Figure S3a better than our selected parameter combination (grey line). However, such a higher fluoride adsorption/desorption ratio leads to lower surface potentials and thus misses the reported value of 70 mV, as one can see from Table S1. Additionally, with the increased fluoride adsorption/desorption ratio, the flow-induced changes can no longer be described sufficiently. The first regime of a flow-induced increase is missed completely, and the flow-induced decreases are overestimated. A similar worsening is achieved when increasing the

adsorption/desorption rate of calcium. In contrast, when decreasing the adsorption/desorption rates, the model does hardly show the regime of flow-induced decreases of the v-SFG intensity and overestimates the flow-induced increases significantly.

One may also consider modifying both adsorption rates in order to yield a better description. In fact, there is no combination that could improve the description of flow-off intensities, flow-induced changes, and surface potential. For instance, the decrease in the surface potential when increasing the fluoride adsorption/desorption rates could be compensated by increasing the calcium adsorption rate. However, both changes in the adsorption/desorption rates worsen the description of the flow-induced changes in the same direction and will therefore not compensate for each other (dark red and light blue lines in Figure S3b).

The purpose of the modeling is to estimate the ratio between the fluoride and calcium adsorption/desorption rates: With the used parameters, the ratio of the rate constants for fluoride and calcium adsorption/desorption differ by about five orders of magnitude. The variation in the parameters by a factor of only 5, changes the surface potential substantially and worsens the description of the flow-induced changes in the v-SFG intensity significantly. Therefore, and given the simplicity of the model, we are confident in concluding from the modeling that the fluoride adsorption/desorption ratio is several orders of magnitude larger than the calcium adsorption/desorption ratio. This clear dominance makes it remarkable that the small contribution of calcium adsorption/desorption can determine the flow-induced changes at a certain fluoride bulk concentration. These observations highlight the sensitivity of our experiments to different adsorption/desorption reactions. Clearly, one needs a very sensitive experimental approach, like the combination of surface-specific v-SFG spectroscopy and flow experiments, in order to uncover the complex interplay of such reactions.

S4 Estimation of Surface Charges

Though treating the extracted values of the adsorption/desorption rate constants with caution, we would like to further discuss the switch in the potential determining role of fluoride and calcium ion adsorptions as well as the range of surface charges where the observed effect occur.

To get a feeling for the switch in the potential determining role, we first consider the isolated adsorption processes. Mathematically, we introduced the surface charge as a function of the interfacial fluoride concentration in section S1 leading to equation (S-8). Similarly, one can derive an equation for calcium adsorption/desorption only based on equation (S-9) leading to equation (S-18). With those equations and the adsorption rates from our model, $k_{\mathrm{ads,F}}/k_{\mathrm{des,F}} = 700$ m³·mol⁻¹ for fluoride and $k_{\mathrm{ads,Ca}}/k_{\mathrm{des,Ca}} = 0.005$ m³·mol⁻¹ for calcium adsorption/desorption, we compare the surface charges resulting from the two individual adsorption/desorption processes in Figure S4a. It is clear from equation (S-8) that the maximum surface charge based on fluoride adsorption/desorption is $1.6\ \mathrm{A \cdot s \cdot m^{-2}}$ $(e\,\Gamma)$ at $c_{\mathrm{F}} = 0$ mM. From this value there is a rapid decline of the surface charge with fluoride concentration upon fluoride adsorption. In contrast, there is a small rise in surface charge with calcium concentration in Figure S4a. However, the rise in surface charge with the calcium concentration is persisting whereas the surface charge as a function of fluoride concentration converges to zero. Therefore, when both reactions are considered, the strong fluoride adsorption leads to an overall reduction of the magnitude of the surface charges when the fluoride concentration increases as we can see in the static v-SFG intensity. But the differential, as we probe it by the flow experiment, can eventually be determined by the calcium reaction, which explains the switch in the sign within the flow experiment.

$$\sigma = \frac{2e\,\Gamma}{1 + \frac{k_{\mathrm{des,Ca}}}{k_{\mathrm{ads,Ca}}}\frac{1}{c_{\mathrm{Ca}}}} \qquad \text{(S-18)}$$

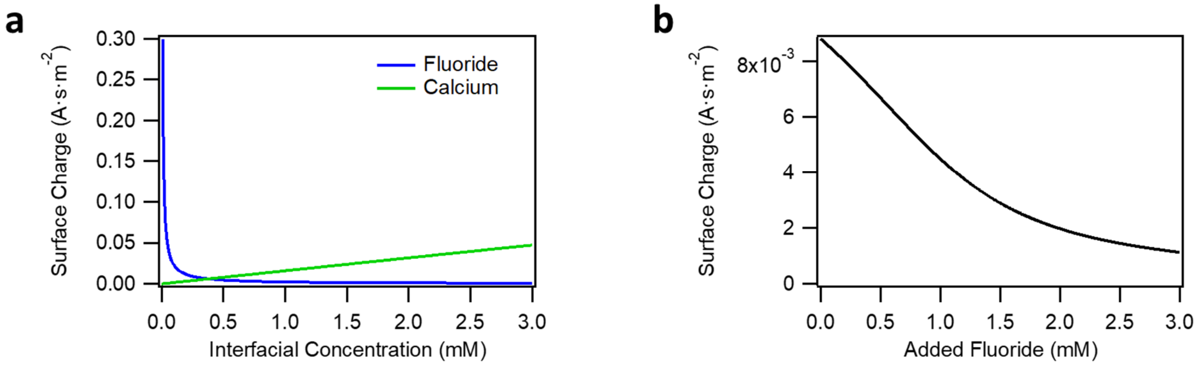

Figure S4 Evaluation of estimated surface charges a) Surface charge as a function of isolated fluoride (blue) and calcium (green) adsorption according to equations (S-8) and (S-18), respectively. b) Surface charge as a function of added fluoride with the concentrations of fluoride and calcium shown in Figure 2 of the main text. The curve is based on equation (S-15).

Finally, we would like to comment on the surface charge range within our experiment considering the concentrations of both fluoride and calcium according to Figure 2 of the main text and the combination of the two adsorption reactions as in equation (S-12). In Figure S4b we show at which

surface charge ranges our experiments are estimated to operate. Based on the introduced maximum surface charge in the range of $1\ \mathrm{A} \cdot \mathrm{s} \cdot \mathrm{m}^{-2}$, we can conclude that less than 1% of the surface sites are charged. Therefore, the observed changes occur on a comparably small range of involved surface sites. This can be explained by the fluoride adsorption that dominates the magnitude of the surface charge via covering the surface. The fact that the estimated range of surface sites in which the observed changes occur is rather low, stresses again the sensitivity of our experimental approach.

S5 Methods

V-SFG spectroscopy. The v-SFG data presented in this work are recorded with an experimental setup that has been described previously.[3] The spectra were recorded in total internal reflection geometry and are not normalized to the IR shape because it is challenging to record a reliable reference spectrum of the IR after the transmission through the prism.[2] Additionally, to investigate the change in v-SFG intensity and surface charge for different fluoride bulk concentrations, such normalization is not necessary. All spectra are recorded in ssp polarization combination (s-polarized SFG, s-polarized visible, and p-polarized IR) and at incident angles $\theta_{vis} \approx 74°$ and $\theta_{IR} \approx 80°$ of the visible and infrared pulse, respectively. Those angles varied between the conducted experiments over some degree due to changes in the alignment. However, within one series of measurements at one day (e.g. going once through all fluoride concentrations), the alignment was not touched after initial optimization. The normalized intensities (i.e., Fig. 1b of the main text), as well as the flow-induced changes (i.e., Fig. 2b of the main text) as function of the concentration, are averaged over several series (days). Direct comparisons (i.e., Fig. 1a and Fig. 2c of the main text) are between data from the same day.

Chemicals. Sodium fluoride was purchased from Merck (99.99%), calcium dichloride dihydrate from Sigma (99%), and concentrated hydrochloric acid from VWR (37 w%). All reagents were used as received without further purification.

Sample preparation. The rectangular fluorite prism (dimensions 4 cm × 1 cm × 0.5 cm) purchased from EKSMA optics was baked at 500 °C for at least 2 h to remove organic residues. Before and after baking, it was rinsed with demineralized water, filtered with a Millipore unit (resistivity = 18MΩ cm). The salt was added to a 1 mM HCl solution, which was prepared by volumetric diluting concentrated acid (37 w%) with Milli-Q water. Usually, a concentration series was prepared via a volumetric dilution series.

Flow setup and experimental procedure. The flow setup and the experimental procedure has been described previously.[3] Spectra were recorded at the center of the flow channel. The flow rate was ~6 $mL \cdot min^{-1}$, corresponding to a Reynolds number of ~25 and a shear rate of ~9 s^{-1}, ensuring laminar flow. A reservoir with ~100 mL of the solution is connected with the flow cell via Tygon® tubes (ISMCSC0832 MHSL 2001; inner diameter 4.8 mm, outer diameter: 8.0 mm). The flow channel as described in Ref. 3 has a volume <1 mL. A homemade LabView program controls the peristaltic pump (Masterflex 77924-70 L/S Ethernet/IP Network-Compatible Pump). For the flow-off intensities at different fluoride concentrations, we waited 10 min after introducing the solution before measuring to ensure equilibration of the surface charge and took 10 s spectra. For the flow experiments, we waited 5 min before recording the first flow-off spectrum of the series of spectra and took 5 s spectra.

Data processing. The spectra were processed by a self-written MatLab script, which has been described previously.[3] From that script, we obtained time traces of the v-SFG intensity, as shown in Fig. S5. Since experimental parameters such as laser power or alignment may change between different days, the pH 3 intensity is measured every day, and referencing the data to its intensity allows a day-to-day comparability of the intensities. In some experiments, the v-SFG intensity shows a long-term slope, on top of which the flow-induced changes occur. This long-term development is not correlated to the investigated fluoride concentration, as shown in Fig. S5a and b, where it is only present in Fig. S5b, although both experiments were conducted with the same sodium fluoride concentration. Moreover, this slope can be both positive and negative, i.e., an increase/decrease in

intensity as in Fig. S5b and Fig. S5c, respectively. Since the underlying process seems to be unspecific, and not reproducible, we assign it to slow drifts in the laser intensity. However, long-term processes that change the surface structure or reactivity might also be an explanation. In order to disentangle the unspecific development from the flow-induced changes, that we aim to investigate, a linear correction of the intensities was applied. This correction is done by aligning the flow-off states before and after the flow-on period, as shown in Fig. S5b and c. After the correction, if necessary, relative changes of the signal upon flow are calculated by averaging spectra over ~1 minute prior to turning flow on and off as shown in Fig. S5a. The flow-induced changes of the different cycles (i.e., two cycles in Fig. S5a) and of different days (e.g. Fig. S5a and b) are averaged.

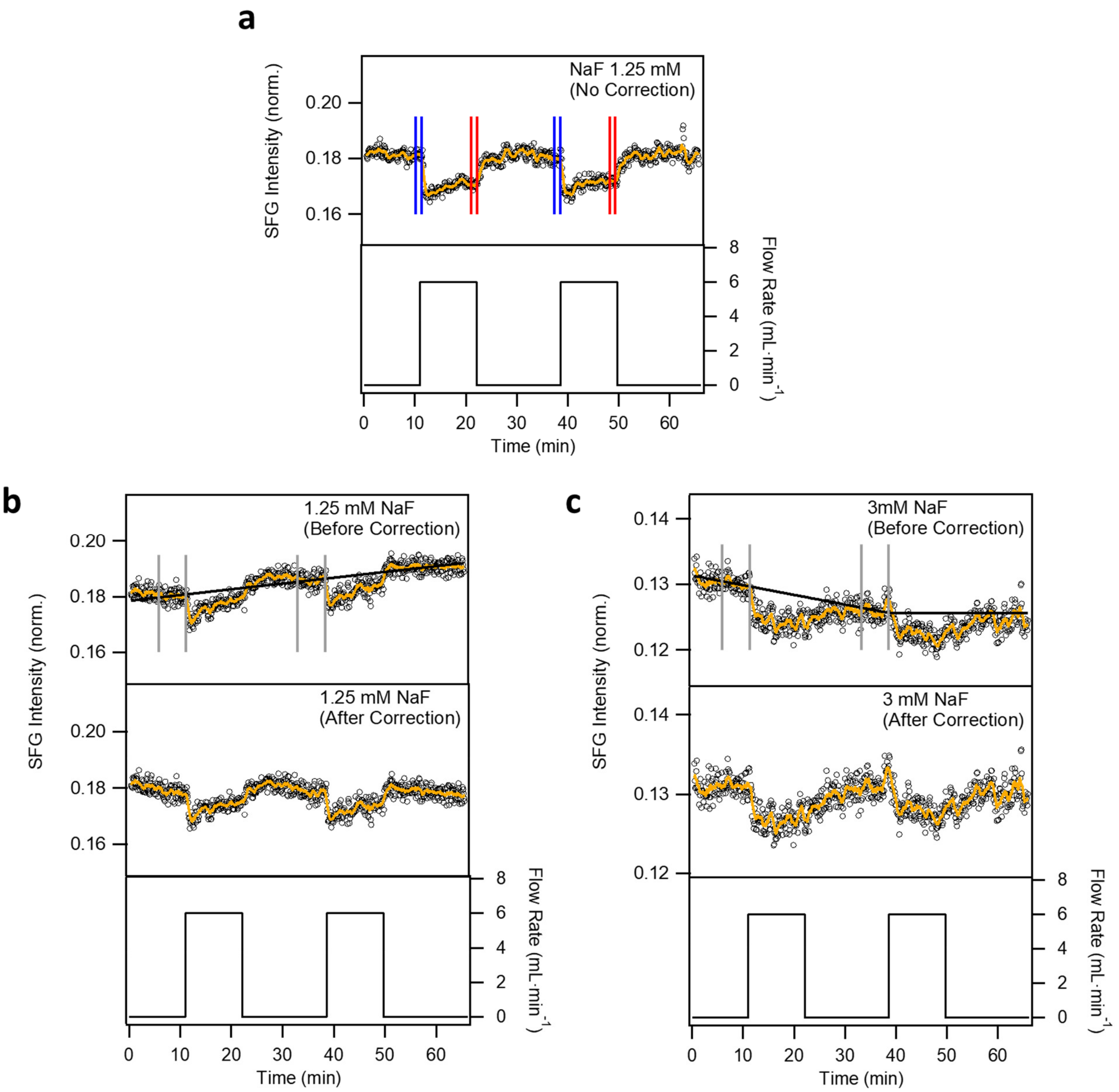

Fig. S5 Remarks on data processing. V-SFG intensity development during two flow-on/off cycles for a calcium fluoride prism in contact with a 1 mM HCl solution and an added amount of sodium fluoride, as indicated in the figure. Each open black circle corresponds to a spectrum that was integrated between 2800 and 3600 cm^{-1}. The colored solid lines are ten-point moving averages to guide the eye. The bottom panels show the applied flow rates (black solid lines) a) The intensities of the flow-off regimes reach the same level. Therefore, no long-term effect seems to be present. The intensity trace was not corrected. The blue and red lines highlight the regions at the end of a flow-off and flow-on interval, respectively. Those regions were taken to calculate relative changes upon flow as in Figure 2b of the main text. b) The intensities of the flow-off regimes do not reach the same level (top). A linear correction between the first two flow-off intervals was applied. The grey lines highlight the regions at the end of the flow-off intervals that were used for a linear fit (black solid line) that corrects for the underlying increase over the whole experiment that we are correcting for. The corrected intensity trace is shown in the middle panel. c) Same as a), but this time there was an underlying decrease which is only present until the second half of the flow-on/off experiment.

Supporting Literature

1 John R. Rumble, e. *CRC Handbook of Chemistry and Physics*. (CRC Press/Taylor & Francis, Boca Raton, FL, 102. ed. Internet Version 2021).

2 Lis, D., Backus, E. H. G., Hunger, J., Parekh, S. H. & Bonn, M. *Science* **344**, 1138-1142 (2014).

3 Ober, P. *et al. Nat. Commun.* **12**, 4102-4112 (2021).

4 Miller, J. D., Fa, K., Calara, J. V. & Paruchuri, V. K. *Colloids Surf. A* **238**, 91-97 (2004).
